# Erysipelas of Newborn Children

**Published:** 1847-03

**Authors:** 


*Erysipelas of Newborn Children* By Prof. Trousseau.—This is one of the most dangerous maladies which can affect the newborn child, not only in hospitals but in private practice; it is almost invariably fatal, particularly during the first month of extra-uterine life. Its danger gradually decreases as the child grows older; but still, after the fourth month, about one-half of the infants affected with it sink under its symptoms. The first appearance of this malady is treacherously insignificant: the child has merely lost a little of his good humour; his sleep is slightly diminished, and he sucks rather less than before; at the same time the skin of the pubes is the seat of a small red patch, painful to pressure; the redness gradually gains in extent on the body and limbs, and is occasionally disseminated; but when, in their turn, the foot and hand become affected, they acquire a degree of swelling and redness far greater than that assumed by the eruption in any other region. The genital organs sometimes sphacelate, in consequence of the local inflammation, and in many cases acquire an emphysematous appearance. The pubes is not the only part from which the erysipelas may take its departure: it has been observed to arise from the redness which surrounds the vaccine pustule, less frequently from any accidental laceration of the skin, or from any one of those divisious so common in the inguinal or other cutaneous folds. The general symptoms are interesting in many respects, and amongst others, because their mildness almost inevitably leads the unwary to commit errors of prognosis, which in private practice are neither forgotten nor forgiven. At first the disease appears perfectly local; it is not until several days have elapsed that general uneasiness and crossness show themselves. The colour of the skin, the expression of the countenance, often remain for some days perfectly satisfactory, when suddenly an ashy cadaverous paleness is observed, and with that degree of intensity that the child seems to be sinking under copious hemorrhage. The cry becomes incessant, jactitation continual, and loss of sleep absolute. These signs are followed by deep stupor and death; the pulse is at first frequent, and the heat of the skin ceases only during the terminal and fatal stupor. Convulsions, diarrhœa, and vomiting are very seldom met with. When the progress is such as we have described, peritonitis has occurred—a frequent disease in children, and one which has not been hitherto described. The duration of the disease varies considerably, sometimes being frightfully short, at others, on the contrary, being prolonged so far as three weeks.

On *post-mortem* examination the cutaneous alterations are occasionally the only changes observed; but when peritonitis (a frequent complication) has been present, the umbilical vein is often found inflamed and filled with pus as far as the transversal furrow of the liver, and inflammatory secretions are found on the peritoneal surface of the abdominal viscera.

Of the probable cause of the malady we may say this much, that we observe it principally when an ill wind of puerperal fever blows over the lying-in hospitals of Paris. The children seem to have inherited from their mother a purulent diathesis, and they appear still to be within certain limits subject to the same ailments as the mother, whose constitution has so lately been theirs. The peritonitis of the children may be, therefore, as aptly termed puerperal as that of the mother, because its general cause is to be sought for in the circumstances which have accompanied the last stage of child-bearing and parturition. It is natural that the skin should be the seat of disease, because that surface has been so lately called to perform functions as new as they are important; in such children the unbilical cicatrix does not form readily, and is sometimes the occasional, the local, cause of the cutaneous disturbance. With regard to the treatment, we have tried almost every local application imaginable, and without success; ointments, lotions, blisters—even the actual cautery—without suspending the progress of the disease. Three cases only have we seen recover during the first month under the use of the ethereal solution of camphor, and of baths containing corrosive sublimate; but in how many have we not since tried the same methods without the slightest benefit! —*London Medical Times.*

